# Compromised functionality of monocyte-derived dendritic cells in multiple myeloma patients may limit their use in cancer immunotherapy

**DOI:** 10.1038/s41598-018-23943-w

**Published:** 2018-04-09

**Authors:** Prajakta Shinde, Sophia Fernandes, Sameer Melinkeri, Vaijayanti Kale, Lalita Limaye

**Affiliations:** 1 National Centre for Cell Science, NCCS Complex, Savitribai Phule Pune University Campus, Ganeshkhind, Pune, 411007 India; 2 Blood and Marrow Transplant Unit, Deenanath Mangeshkar Hospital, Erandawne, Pune, 411004 India

## Abstract

Dendritic cells (DCs) have the potential to elicit long-lasting anti-tumour immune responses. Most of the clinical trials of anti-cancer DC vaccines are based on monocyte-derived DCs (Mo-DCs). However, their outcomes have shown limited promise especially in multiple myeloma (MM) patients. Here, we investigated whether *in vitro* generated Mo-DCs from MM patients (MM-DCs) possess impaired functionality, thus contributing to the limited success of DC vaccines. We generated MM-DCs and compared them with DCs from healthy donors (HD-DCs). The yield of DCs in MM was 3.5 fold lower than in HD sets. However morphology, phenotype, antigen uptake and allo-T cell stimulation were comparable. Migration and secretion of IL12p70 and IFN-γ (in DC-T cell co-cultures) were significantly reduced in MM-DCs. Thus, MM-DCs were compromised in functionality. This impairment could be attributed to autocrine secretion of IL6 by MM-monocytes and activation of their P38 MAPK pathway. This indicates a need to look for alternative sources of DCs.

## Introduction

Dendritic cells (DCs) play a pivotal role in the immune system by orchestrating T cell immune response. They capture, process and present antigens to T cells. Interactions of DCs with other immune cells like NK cells^1^, B cells^2^ and macrophages^3^ are also very well known. Owing to their immune regulatory properties, they are used as cancer vaccines. DCs loaded with tumor- associated antigens can act as inducers of antitumor T cells, which can ultimately lead to tumor regression^4^.

Multiple myeloma (MM) is a malignancy of plasma cells differentiated from B cells. These cells continue to secrete immunoglobulin, which accumulates in the bone marrow and form lesions, thus hindering normal haematopoiesis. Although treatments such as stem cell transplantation (SCT) and chemotherapy have increased the progression-free survival in multiple myeloma patients, they often undergo relapse. Monoclonal antibodies and chimeric antigen receptor (CAR) -T cells against idiotype protein secreted by tumor cells provides an option for immunotherapy, but it doesn’t impart immunological memory to prevent a relapse^5^. On the other hand, DCs, when used as a vaccine, induce long-lasting anti-tumour immune responses through effector and memory T cells in the body^6^. Thus, DCs hold the promise for use in the treatment of multiple myeloma.

The absolute count of DCs and their precursors circulating in the peripheral blood in MM patients is known to be decreased^7^ and they are also immunologically compromised^8,9^. As DCs from cancer patients can not be directly used for vaccine preparation, *in vitro*-differentiated monocyte-derived dendritic cells (Mo-DCs) are commonly used as an alternative.

Phase I and Phase II clinical trials of Mo-DCs loaded with tumor-associated antigen (TAA) used as a cancer vaccine have provided evidence of safety in various types of cancers, such as melanoma, prostate cancer, malignant glioma, renal cell carcinoma and MM^10^. However, though growing numbers of preclinical studies have shown that DC vaccines have the potential to be used as a therapeutic modality; sufficient responsiveness has not been seen in many clinical trials. The efficacy of the Mo-DC vaccine especially for MM treatment is still suboptimal, with varying degrees of success^11–14^. It is likely that the lack of responsiveness to Mo-DC vaccines is partly due to defects in these DCs. Since *in vitro* differentiated Mo-DCs from multiple myeloma patients are poorly studied for their phenotype and functionality, there is a need for a systematic evaluation of these DCs. In order to know whether Mo-DCs from MM patients possess diminished immune functions, we compared morphology, phenotype and functionality of *in vitro* generated Mo-DCs from MM patients (MM-DCs) with Mo-DCs from healthy donors (HD-DCs). We report here that monocyte-derived DCs from MM patients are indeed defective in migration and secretion of key cytokines. Autocrine secretion of IL6 and activation of the P38 MAPK pathway probably contribute to impaired migration of MM-DCs.

## Results

### Though morphology and phenotype of HD-DCs and MM-DCs were similar, cell yields were drastically different

**Figure 1:**
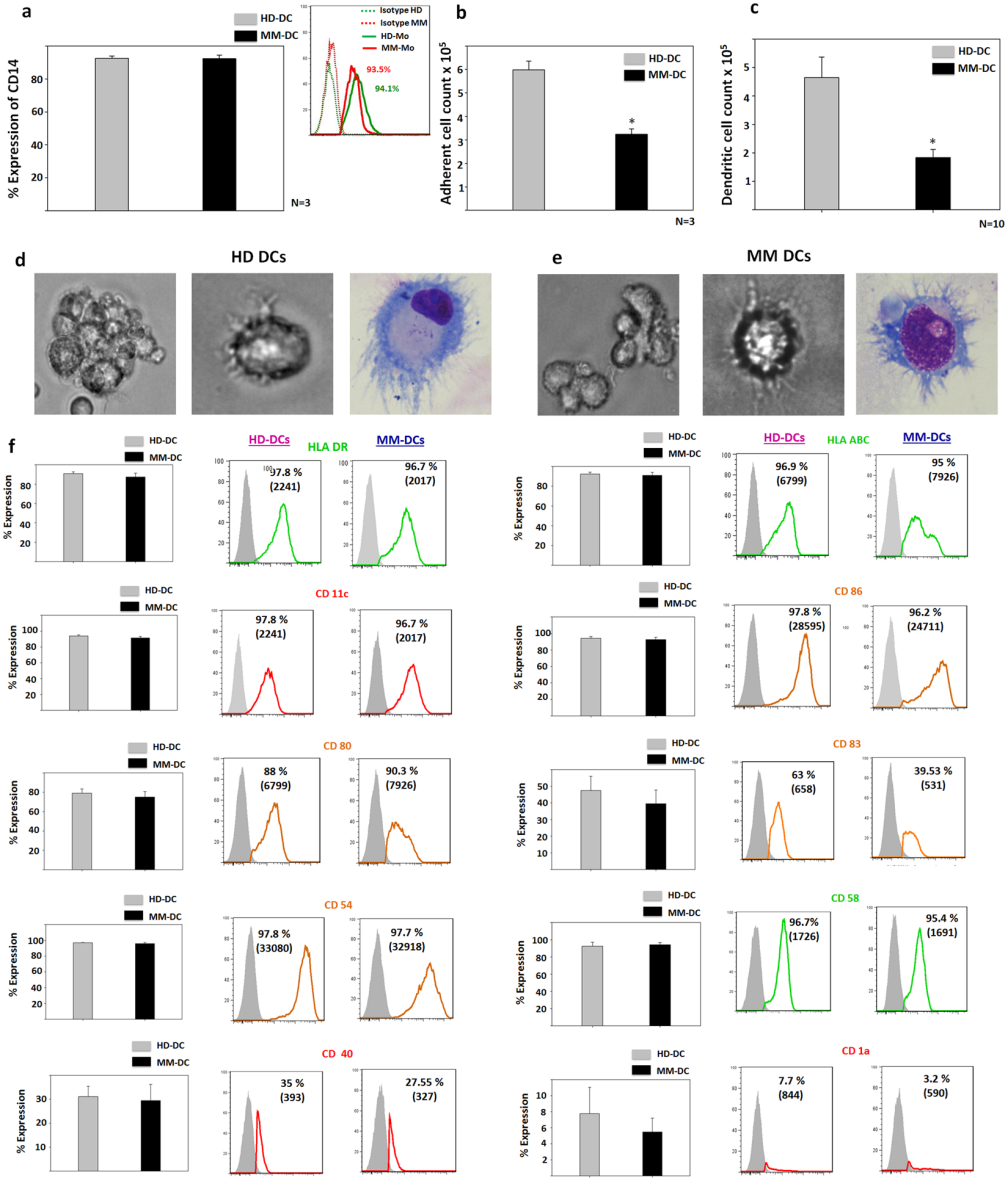
Cell yield, Morphology and phenotype of HD-DCs and MM-DCs: Quantitative data showing (**a**) Percent expression of CD14 on gated MNCs (N = 3) (**b**) No. of adherent cells obtained from 10^7^ MNCs of HD and MM samples (N = 3, p ≤ 0.05*). The experiments were performed on three HD and three MM samples (N = 3) with triplicates (n = 3) of each sample (**c**) Absolute number of DCs (N = 10, p ≤ 0.05*). Phase contrast images of DCs generated from (**d**) HD-DCs and (**e**) MM-DCs at 10X (left) and 20X (Middle) of magnification. Wrights-Giemsa stained images of DCs from HD-DCs and MM-DCs respectively at 20X (right) magnification. (**f**) Flow cytometric assessment for the expression of surface markers confirms that DCs have mature phenotype. The experiment was performed on 10 different HD and 10 different MM samples (N = 10). The data shown are mean ± SEM.

The mononuclear cell (MNC) population from HD and MM samples were analysed for expression of CD14 to test if there was a difference in the monocyte marker expression. The MNCs from both the samples showed similar expression of CD14 (Fig. 1a). DC cultures were then established from adherent monocytes, after seeding equal number of MNCs as described earlier and the viable cells in the adherent fraction were taken. While the viability of adherent cells from HD and MM was similar, the count of adherent cells in the MM samples was significantly lower than the HD samples (Fig. 1b). This difference in the precursor cell count was also reflected in the DC count. The DC yield from healthy samples was significantly higher (3.5 fold), as compared to MM samples, when 10^7^ MNCs were seeded for adherence Fig. 1c). As MM samples had low DC precursor population, the yield of DCs obtained from these samples was also low.

Observation of the DCs under a phase contrast microscope showed adherent as well as non-adherent clusters. Cells with typical stellate processes and veiled appearance were observed. Differential staining of cells with Wrights-Giemsa stains revealed typical dendritic morphology with dense cytoplasm and nucleus. DCs from both the HD and MM samples appeared similar (Fig. 1d and e).

Flow cytometry analysis for expression of surface markers on both HD-DCs and MM-DCs showed mature phenotype with very high expression (>90%) of the MHC class I molecule HLA-ABC, the MHC class II molecule HLA-DR, the DC-specific molecule CD11c, costimulatory molecules CD86, CD80 and CD83, integrins and adhesion molecules like CD54 and CD58. The expression of these markers above 80% in HD-DCs and MM-DCs. Expression of the costimulatory molecule, CD40, was lower in the MM-DCs (31.15% ± 4.3 and 29.13% ± 6.8 for HD-DCs and MM-DCs respectively). Expression of the MHC class-like molecule, CD1a, was very low (about 4%) in both DCs. The marginal difference in the percent expression as well as MFI of these molecules in HD-DCs and MM-DCs did not reach statistical significance (Fig. 1f). Thus, the data shows that HD-DCs and MM-DCs have mature phenotype.

### HD-DCs and MM-DCs were equivalent in antigen uptake capacity

**Figure 2:**
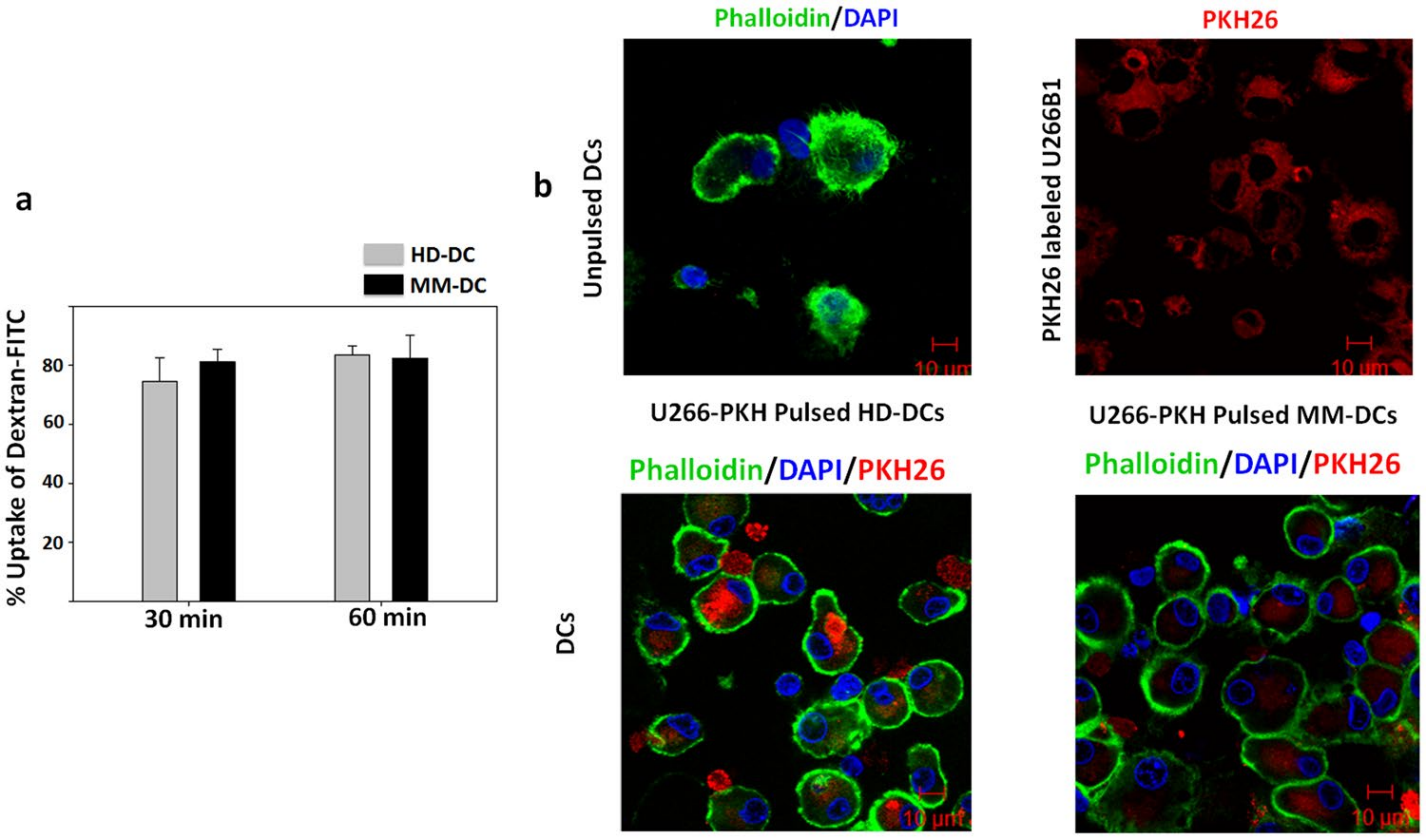
Antigen uptake HD-DCs and MM-DCs was similar: Immature HD-DCs and MM-DCs were compared for the extracellular antigen uptake capacity. (**a**) Uptake of Dextran-fluorescein isothiocyanate (FITC) at 30 min and 60 min of incubation at 37 °C is shown. The experiments were performed on three HD and three MM samples (N = 3, mean ± SEM). (**b**) Confocal microscopy showing uptake of fluorescently labelled U266B1 cell lysate, by HD-DCs and MM-DCs. DCs were labelled with Phalloidin-FITC (green) and their nuclei with DAPI (blue). Upper left panel show control set of DCs without any addition of fluorescently labelled U266B1 lysate. Upper right panel show Multiple myeloma cell line U266B1, fluorescently labelled with PKH26 (Red). Lower left panel shows test HD-DCs and right panel show test MM-DCs that had internalised fluorescently labelled U266B1 cell lysate.

Loading of DCs with extracellular antigens is an important step during DC vaccine preparation. In their immature state, DCs take up extracellular antigens via pinocytosis and receptor-mediated endocytosis. We assessed the receptor- mediated endocytosis function of DCs using the fluorescently-labelled Dextran (Dextran-FITC) uptake assay. The antigen uptake was very high (74.5% ± 7.961 in HD-DCs and 83.5% ± 3.09 in MM-DCs) at 30 mins and 60 mins time points in both the DCs (Fig. 2a). Thus, DCs from both the sources had equivalent capacity to uptake extracellular antigen via receptor mediated endocytosis.

To further confirm the uptake of tumor-associated antigens, we used the human multiple myeloma cell line, U266B1 which was labelled with the PKH26 dye to emit red fluorescence. Following administration of the labelled cell lysate to immature HD-DCs/MM-DCs, internalisation of tumor antigens was observed as red fluorescence in DCs, under a confocal microscope. The uptake appeared similar in HD-DCs and MM-DCs (Fig. 2b). Thus we conclude that there was no significant difference in the antigen uptake capacity of the HD-DCs and MM-DCs.

### T cell stimulatory ability of both HD-DCs and MM-DCs was equivalent

DCs have the characteristic function of inducing proliferation of allogeneic-T cells. We performed co-cultured HD-DCs and MM-DCs with sort-purified and CFSE-labelled CD3 + T cells from allogeneic healthy donors. After 5 days of co-culture, we observed an increase in the absolute counts of T cells, as compared to T cells incubated without DCs.

**Figure 3:**
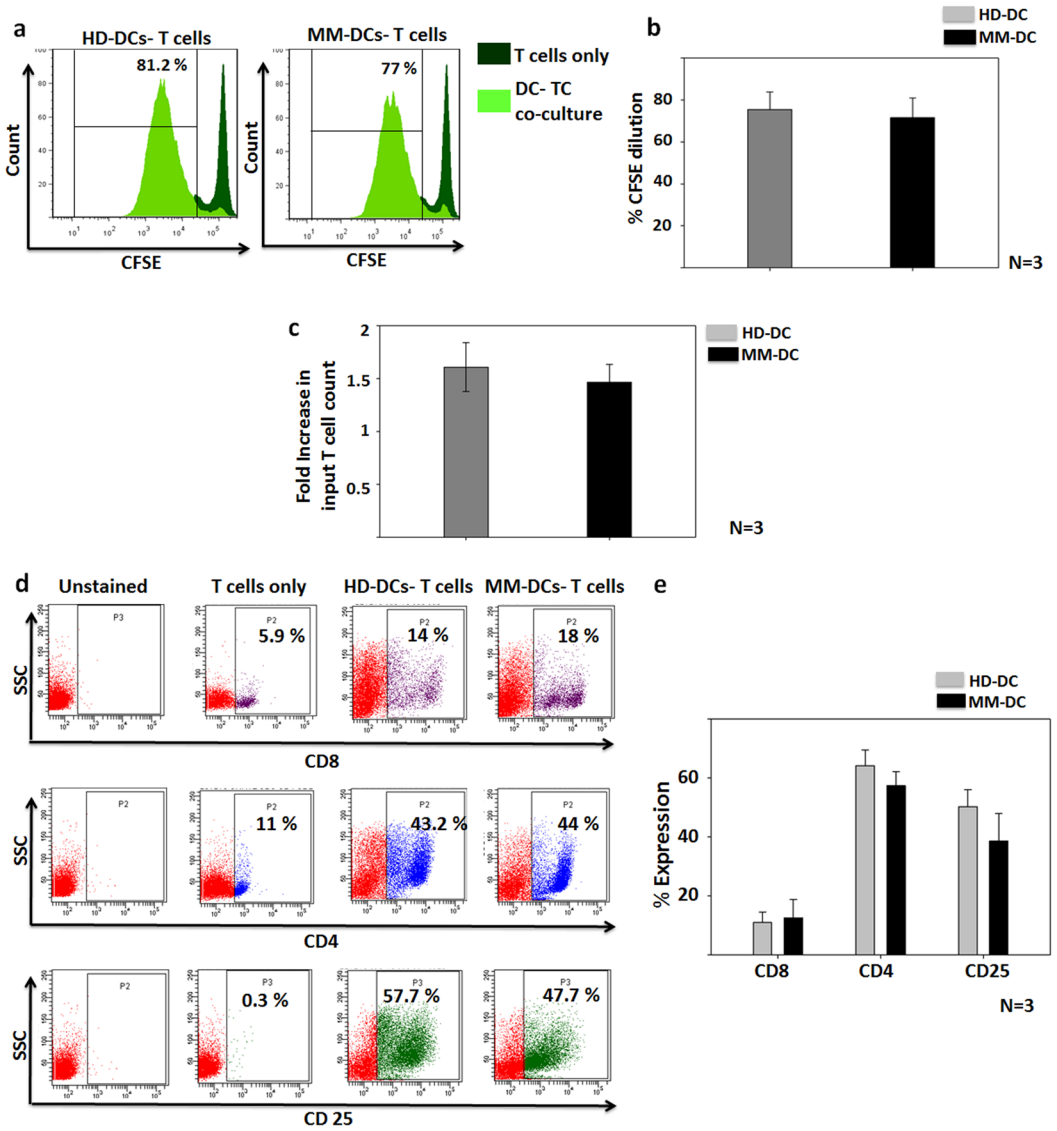
Allogeneic T cell proliferation in co-culture with HD-DCs and MM-DCs was equivalent: HD-DCs and MM-DCs were assessed for their ability to induce proliferation of allogeneic T cell from healthy volunteers (**a**) Dilution of CFSE in HD-DCs and MM-DCs in 5 days co-cultures, overlays of one representative experiment is depicted. (**b**) Percent of CFSE dilution in both cultures from three independent experiments is depicted. (**c**) Quantitative data showing increase in live T cell count, on day 5 over input cells is shown. (N = 3) (**d**) Flow cytometry dot-plot for CD8+, CD4+ and CD25+ T cells in the 5 days culture of T cells only; HD-DCs-T cells and MM-DCs-T cells of one representative experiment. (**e**) Cumulative percent of CD8, CD4 and CD25 in both co-cultures (N = 3). The data shown are from three HD and three MM samples (N = 3). The experiments were performed with each sample in triplicates (n = 3). Mean ± SEM values are given.

In both HD-DCs and MM DCs co-cultures, T cells showed CFSE dye dilution, which is indicative of their proliferation (Fig. 3a). Though the proliferation (75.35% ± 8.38 in HD-DCs and 71.5% ± 9.425 in MM-DCs, Fig. 3b) as well as the fold increase of T cells count (1.6 ± 0.23 fold in HD-DCs and 1.46 ± 0.166 fold in MM-DCs, Fig. 3c) was lower in MM-DCs, as compared to HD-DCs, the difference was marginal. It is noteworthy that, there was no difference in the proliferation of CD8+ and CD4+ subsets of T cells in the two sets, suggesting that there was no functional deviation in T helper (CD4) and cytotoxic T cell (CD8) subsets. Though the expression of CD25, which represents activated T cells, was less in MM-DCs as compared to HD-DCs co-cultures, the difference was not statistically significant (Fig. 3d shows representative data and Fig. 3e shows data from 3 samples). Collectively, these data indicate that HD-DCs and MM-DCs are equivalent in their ability to induce proliferation of allogeneic T cells.

### Cytokine profiles of the two DCs sets and DC-T cell co-cultures were different

**Figure 4:**
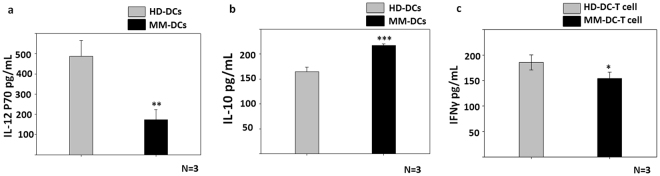
HD-DCs or MM-DCs showed different IL-10 and IL-12p70 secretion profile, IFNγ in MM-DCs and T cell co-culture was reduced: (**a**) Detection of IL-12p70 (N = 3) (**b**) IL-10 in the culture supernatants of HD-DCs and MM-DCs (N = 3) (**c**) Supernatants from allogeneic HD-DCs-T cells and MM-DCs-T cells co-cultures were analysed for amount of IFNγ by ELISA (N = 3). The data shown are mean ± SEM. p ≤ 0.05*, p ≤ 0.01**, p ≤ 0.001***. The experiments were performed on three HD and three different MM samples (N = 3) with triplicates (n = 3) of each sample.

DCs should have high levels of IL12p70 and low levels of IL10 cytokine secretion to evoke a good Th1 response. Hence we compared the cytokine profiles of HD-DCs vs MM-DCs. We found significant differences in the cytokines secreted by HD-DCs and MM-DCs. Lower levels of IL12p70 (Fig. 4a) and higher levels of IL10 (Fig. 4b) were observed in the MM-DC supernatant, as compared to the HD-DC set. As expected, we observed significantly lower Th1 cytokine IFNγ secretion in the supernatant of MM-DCs-T cell co-cultures as compared to HD-DCs-T cells co-cultures (Fig. 4c). These cumulatively indicate that, the T cells that are co-cultured with MM-DCs may not be as immunogenic as those co-cultured with HD-DCs.

### MM-DCs exhibited impaired migration as compared to HD-DCs

**Figure 5:**
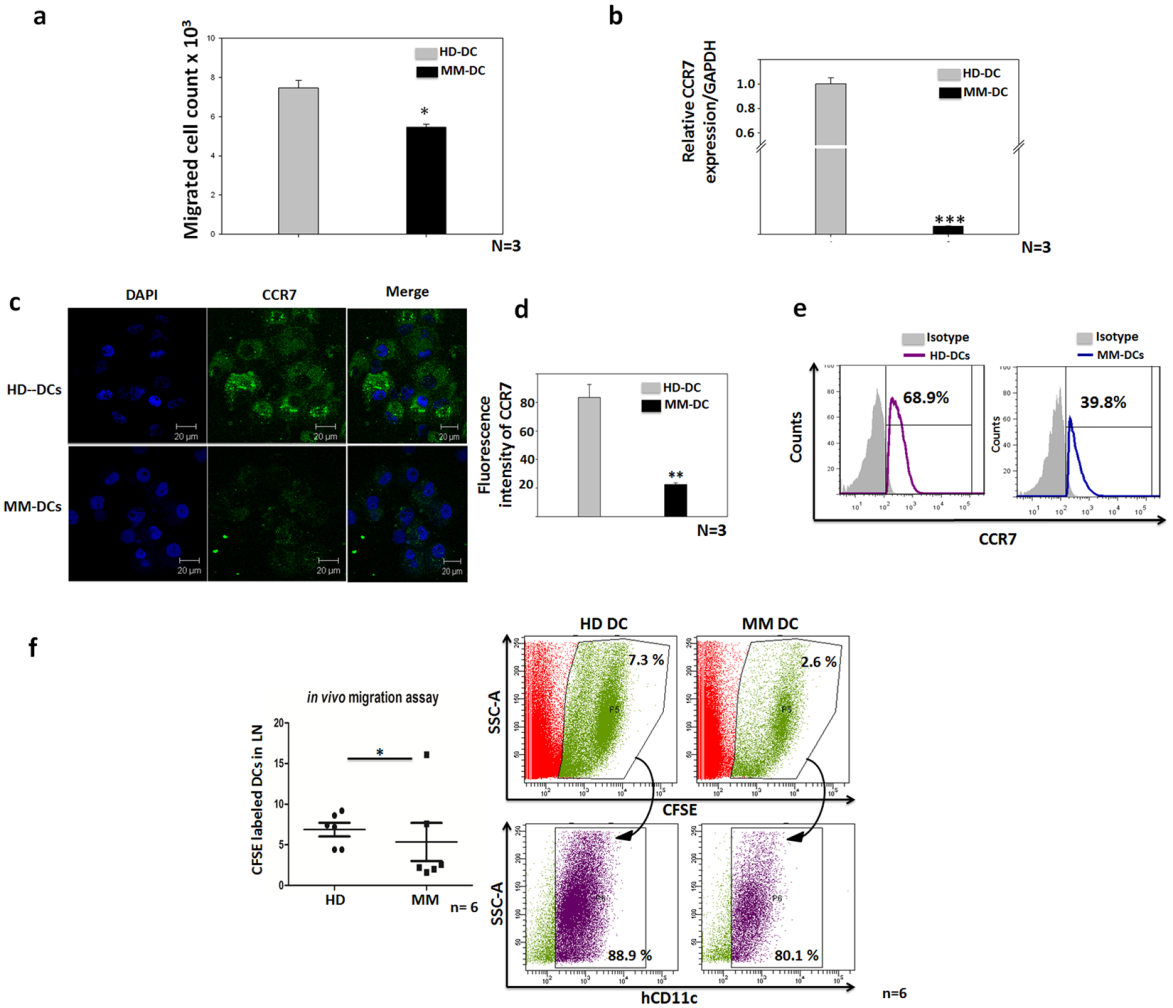
MM-DCs show impaired migration as compared to HD-DCs: (**a**) *In vitro* migration of DCs to CCL19. The experiments were performed on three HD and three MM samples (N = 3) with triplicates (n = 3) of each sample. Data from one representative sample is shown (n = 3). Individual values for each sample are given in Supplementary Table S2. (**b**) Relative CCR7 mRNA expression normalized to GAPDH (N = 3). (**c**) Immunofluorescence (IF) for detection of CCR7 expression on HD-DCs and MM-DCs. (**d**) Fluorescence intensity plots shows significantly lower fluorescence intensity of CCR7 on MM-DCs as compared to HD-DCs (N = 3). (**e**) FACS overlays for CCR7 expression of a representative sample of HD-DCs and MM-DCs respectively. (**f**) CFSE-labelled HD-DCs from one sample and MM-DCs from other sample was injected into the groins of NOD-SCID mice (six mice for HD-DCs and six different mice for MM-DCs). *In vivo* migration of DCs towards draining lymph nodes was measured by flow cytometry. Percentages of CFSE population are shown. Typical scatter plots and DCs migration to regional lymph nodes in NOD-SCID mice (left-HD-DCs, right-MM-DCs). The data shown are mean ± SEM, p ≤ 0.05*, p ≤ 0.01**, p ≤ 0.001***.

Under homeostatic conditions, DCs capture antigens from the environment and migrate to the regional lymph nodes to present these antigens to T cells. This migration of DCs is dependent on the C-C chemokine receptor type 7 (CCR7). CCR7 has an affinity towards its ligand, CCL19, which is secreted by cells residing in the lymphatic system. We analysed *in vitro* migration of HD-DCs and MM-DCs through cell culture inserts of 8 µm pore size. The number of HD-DCs that migrated towards CCL-19 was significantly greater than MM-DCs (Fig. 5a). We then checked the expression of CCR7 at the gene (Fig. 5b) and protein levels by confocal microscopy (Fig. 5c and d) and by flow cytometry (Fig. 5e). As expected, CCR7 expression was much higher in HD-DCs as compared to MM-DCs. *In vitro* migration data was corroborated by performing *in vivo* migration of DCs. CFSE-labelled HD-DCs and MM-DCs were injected subcutaneously in the groins of NOD-SCID mice and cells were harvested from the regional lymph nodes 48 hrs after injection, as described in materials and methods. Significantly less (2%) migration of dendritic cells to the lymph nodes was observed in the animals which received the MM-DCs, than those that received HD-DCs (7%, Fig. 5f). Human CD11c-specific antibody staining of the lymph node harvest showed that more than 80% of the CFSE-positive cells were CD11c positive, underscoring the fact that these cells were indeed human DCs. These data indicate that monocyte-derived DCs from MM patients were compromised in their ability to migrate to lymph nodes. Reduced CCR7 expression in MM-DCs is likely to be responsible for restrained migration.

### Autocrine Interleukin-6 and p38 MAPK impairs CCR7-dependent migration in MM-DCs

**Figure 6:**
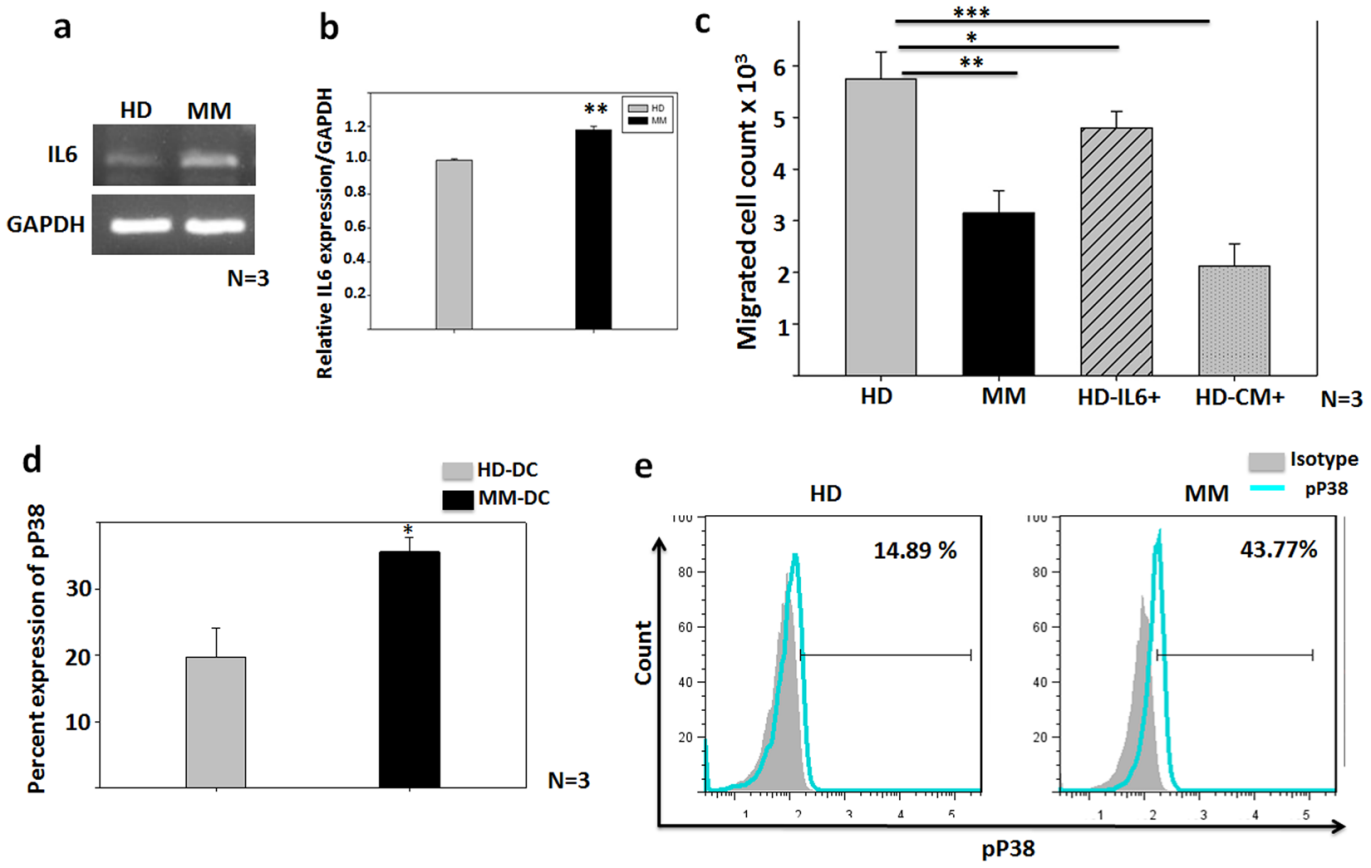
MM monocytes have high autocrine IL6 secretion and activated pP38 pathway: (**a**) mRNA expression of IL-6 and GAPDH on HD and MM samples on day3 of DC culture (N = 3) (**b**) Relative IL6 mRNA expression normalized to GAPDH. (**c**) *In vitro* migration of DCs toward CCL19 is shown of HD-DCs and MM-DCs as controls, test groups are HD-DCs differentiated in presence of external IL-6 or MM cell line CM (N = 3). (**d**) Percent expression of phosphorylated P38 (at pT180/pY182) at day3 of MM and HD monocytes differentiation toward DCs was significantly high (N = 3). (**e**) Representative histogram profile of pP38 on HD (left) and MM (right) day 3 cultures. The experiments were performed on three HD and three MM samples (N = 3) with triplicates (n = 3) of each sample. The data shown are mean ± SEM. p ≤ 0.05*, p ≤ 0.01**, p ≤ 0.001***.

It has been shown that monocytes from MM patients have high levels of autocrine IL6 secretion^15^. In concurrence with this, we found that the mRNA levels of IL6 on day 3 of DC culture was significantly higher in the MM set, as compared to the healthy donor set (Fig. 6a and b). To determine whether high IL6 levels affect the migratory function of DCs, we added exogenous IL6 in the cultures of HD-DCs and observed that IL6-treated HD-DCs behaved similar to MM-DCs. They exhibited impaired migration, as compared to untreated HD-DCs (Fig. 6c), confirming that IL6 does affect the migration of DCs towards CCL19. High levels of IL6 are also associated with poor clinical prognosis for MM patients. Subsequently, we determined the influence of U266B1-derived IL6 on DC migration. We found that the migration ability of HD-DCs was significantly reduced in the presence of conditioned medium from the MM cell line U266B1 as well (Fig. 6c).

In multiple myeloma, another independent pathway, the P38 MAPK pathway is known to be activated in DC progenitors, leading to their dysfunction^15^. We found that monocyte-precursors of DCs had high pP38 MAPK expression in MM samples. This was estimated using flow cytometry analysis of cultures harvested on third day of DCs differentiation (Fig. 6d and e). These data clearly demonstrated that a migration defect in migration capability of MM-DCs was affected through IL6 and activation of the p38 MAPK pathway.

## Discussion

Considered as the master regulators involved in tuning the immune response, DCs are referred to as the professional antigen presenting cells that are essential for activation of the T cells. High levels of expression of costimulatory molecules (e.g. CD86, CD83, CD80 and CD40 etc.) by DCs are necessary to induce naive T cell activation and memory T cell formation^16^. DCs are thought to play an important role in reactivating the immune system against cancer by inducing T cells to produce tumor specific cytotoxic activity^17^.

Cellular vaccines have been explored during the past decade for immunotherapy. These are based on antigen presenting cells, such as DCs. Since the DCs circulating in the peripheral blood of cancer patients are defective^15^, DCs generated from monocytes *in vitro* are generally used for vaccine preparation. They are loaded with tumor-associated antigens and injected back into the patient to boost T cell reactivity against the tumor.

Clinical trials of Mo-DCs in multiple myeloma patients are reported to have shown limited success. Zahradova *et al*. (2011) and Hobo *et al*.^11^ investigated clinical responses in MM patients injected with Mo-DCs loaded with idiotype protein, but the expected response was not observed^11,12^. Rosenblatt *et al*.^13,14^ carried out fusion of multiple myeloma cells and autologous DCs and used them as a vaccine in Phase I and II trials. Their data revealed a clinical response only in a quarter of the patients. Mo-DCs from patients of MM were reported to have worked better in other studies, where DC vaccine was used in combination with other interventions such as chemotherapy^18–20^. A protocol by Lee *et al*.^21^ for multi-peptide-pulsed DCs was also shown to be effective in *in-vitro* settings^21^. While the available literature collectively suggest that administration of DC vaccines could be useful in the treatment of MM, it is necessary to determine the factors contributing to the limited success observed in clinical trials so far. Our findings reported here provide useful insights into some such factors associated with DCs derived from monocytes of MM patients.

We checked whether the DCs derived from monocytes of MM patients have the same potency as those derived from monocytes of healthy donors. We found that some of their functional attributes, such as antigen uptake and allo-T-stimulatory ability were equivalent. However, the actual numbers of DCs generated from MNCs was drastically different in the HD-DCs and MM-DCs, with 3.5 fold less DCs being obtained from 10^7^ MNCs of MM samples as compared to healthy samples, due to the lower monocytes numbers in those samples.

Optimum T cell activation and proliferation is crucial for tumor regression. IFNγ secreted by activated T cell plays an important role in T cell homeostasis by regulating differentiation and activation of desired Th1 cells, while inhibiting Th2 cells. The MM-DCs displayed a profound defect in the ability to secrete T cell stimulatory IL12p70. As a result, levels of the desired Th1 cytokine IFNγ was drastically low in co-culture of MM-DCs and T cells as compared to the HD-DCs and T cell co-culture. In other words the cytokine profiles of DCs and DCs: T cell co-cultures indicated that MM-DCs would evoke a reduced Th1 response. It is evident from our data that Mo-DCs from MM patients were inefficient in inducing the desirable T cell activation in allogeneic co-culture system. The tumor microenvironment in MM causes both functional and quantitative defects in effector T cells^22,23^. There is a well-established correlation between poor disease prognosis and altered phenotype of T cells in MM^24^. Therefore autologous DCs and T cell co-cultures may not elicit a response similar to allogeneic co-culture. Sequential studies are needed to evaluate MM-DCs for their ability to present tumor antigens to autologous T cells and induction of the Th1 phenotype. Various investigators have used *in vivo* tumor bearing mouse model to compare the antigen presentation, migration and T cell activation potential of DCs^25,26^. Similar *in vivo* mouse model study will be required to compare HD-DCs vs MM-DCs. Given that we found functional impairments in DCs obtained from monocytes of MM patients, we have initiated experiments to check whether these defects could be overcome if the DCs are derived from CD34+ cells instead of from monocytes. Having obtained good leads, we are further confirming our observation with an *in vivo* Xenograft mouse model of multiple myeloma.

For an efficient immune response, lymph node homing of DCs via chemotaxis is crucial^27^. Defective migration of DCs to lymph nodes is one of the major hurdles faced by DC-based immunotherapy^26^. Maturation of DCs occurs under inflammatory conditions, followed by migration of the mature DCs from the site of inflammation towards lymph nodes, where they stimulate T cells, initiating an immune response^28^. Our findings revealed subtle defects in the migration function of MM-DCs, with these showing less *in vitro* migration towards CCL19 than HD-DCs. Studies further extended to an *in vivo* setting also revealed that MM-DCs migration to the regional lymph nodes of mice was impaired. Moreover, lower expression of CCR7 at the gene and protein levels in MM-DCs, may prevent inefficient priming of the T cells in the lymph nodes, resulting in less than favourable outcomes in clinical trials of anticancer DC vaccines.

Using peripheral blood monocytes from healthy donors and multiple myeloma patients to derive DCs, Wang *et al*.^15^ showed that MM patient-derived Mo-DCs are phenotypically and functionally defective. They investigated only a few aspects of DC function, such as DC marker expression and T cell stimulation. However migration which is a crucial function of DCs, was not studied by them. On the other hand, studies on G-CSF mobilised peripheral blood samples from MM and HD samples by Ratta *et al*.^8^ revealed no observable differences between HD-DCs and MM-DCs in their functions of antigen uptake and T cell proliferation, similar to our findings. However their report was also lacking data on the migration of DCs. Since tumor antigen trafficking to the draining lymph nodes is crucial for the anticancer DC vaccine, we carried out detailed studies on the migration of DCs and expression of CCR7. Such studies on DCs derived from monocytes of MM patients have not been reported earlier. It is noteworthy that we observed significant differences in these important parameters. Hence our study points to the possibility that monocytes from MM patients may not be a good source to derive DCs for vaccine preparation.

Cancer cells exert a systemic effect on the myeloid compartment of haematopoiesis^29^, leading to impaired differentiation and function of DCs in cancer patients^30^. It is well documented that high expression levels of IL6^31–33^ and an activated P38 MAPK pathway^34,35^ are responsible for the migration defects of DCs in other types of cancers, for e.g. breast cancer, cervical cancer, ovarian cancer etc. Whether this molecular mechanism of defective migration of DCs also exists in MM samples was not clear. Therefore to test the intrinsic defects in the progenitors of DCs, an early time point of DC culture was selected. We found that MM patient-derived progenitor cells do indeed express higher amounts of autocrine cytokine IL-6 and display an activated p38 MAPK pathway. Multiple myeloma cell line-derived CM was also found to exert similar effects on HD-DCs, ascertaining that the presence of tumor microenvironment alters properties of DCs. There is an intricate cross talk between dendritic cells and malignant plasma cells in MM. DCs are shown to support malignant plasma cell proliferation via RANK–RANK ligand and APRIL^36^. In disease progression from monoclonal gammopathy of undetermined significance (MGUS) to multiple myeloma, DCs have shown to protect tumor cells from direct killing by T cells^37^. All these references support the rationale that the immune response initiated by DCs may vary in the presence of tumor cells. For the development of an effective DC vaccine, a comprehensive understanding of the complex tumor microenvironment present in cancer is required. For complete elimination of cancer, the DC vaccine must induce expansion, activation and infiltration of T cells in the tumor. A combination therapy that overcomes the immunosuppression exerted by cancer, and which can optimally recruit the immune system to completely eliminate the cancer needs to be explored. *Ex vivo* generated bona fide DCs can then create a stimulating milieu for the activation of bystander-DCs, with concomitant activation of effector T cells for complete eradication of the disease^38^.

Our findings have helped elucidate some of the possible defects in monocyte-derived DCs generated from MM patients. It can’t be ruled out that factors like the drugs used for the chemotherapy, tumor burden at the time of sample collection and difference in the age of the HD and MM patients may have contributed to the differences observed. Further studies with a large number of samples from MM patients with age and gender matched healthy donors would help throw more light.

In summary, the yield of DCs generated *in vitro* from monocytes of MM patients was significantly less than healthy donor-derived DCs. Though, they showed similarity in morphology, phenotype, antigen uptake and allo-T cell stimulatory ability, MM-DCs were compromised in their chemotaxis function. The underlying mechanisms associated with this dysfunction were elevated expression of autocrine IL-6 and an activated P38 MAPK pathway in the precursors of the DCs. High levels of IL10 and low IL12p70 production by MM-DCs led to significantly low IFNγ in co-cultures with T cells. These findings suggest that alternative sources such as autologous HSCs-derived DCs^39^ or HLA-matched allogeneic monocyte-derived DCs need to be explored for use in MM immunotherapy in lieu of DCs derived from monocytes of MM patients.

## Materials and Methods

### Ethics approval and Sample collection

Human apheresis samples and peripheral blood collection, processing, format of informed consent forms and all experimental procedures were approved by the Institutional Ethics Committee (IEC of NCCS and Deenanath Mangeshkar Hospital, Pune, India) in accordance with the Declaration of Helsinki.

### Collection of clinical samples

Upon completion of the transplantation of apheresis samples to the patients, the leftover sample from the tubing was procured from the transplantation unit. Therefore the volume of the samples was limited. In addition there was unavoidable cell loss during the sample processing especially after freezing and thawing of the cells. Therefore we could study few parameters like morphology and phenotype with more number of samples (N = 10). Some of the samples had enough number of cells to perform dendritic cell functions like migration, MLR, etc in addition to morphology and phenotype. Numbers of samples used in the experiments are mentioned in the figure legends of each experiment.

#### Healthy donors

10 number of apheresis samples were collected from anonymous healthy donors who were donating cells for allogeneic HCT for MM patients other than those included in this cohort study. Age and gender of the donor is specified in Supplementary Fig. S1.

#### Multiple myeloma patients

14 number of apheresis samples were collected from MM patients who were undergoing autologous SCT. Stage of the disease was diagnosed according to The International Staging System (ISS) for multiple myeloma. MM patients underwent chemotherapy regimens to achieve remission of the disease. Samples were collected after three to six months of chemotherapy when the patients were in remission. MM patient’s age, gender and stage of the disease (at the time of first clinic visit) are specified in Supplementary Fig. S1.

Samples collected from healthy donors and multiple myeloma patients for this study were thus unpaired samples.

### U266B1 cell line culture and collection of conditioned media (CM)

Multiple myeloma cell line U266B1 was procured from NCCS cell repository. The cell line was cultured in RPMI-1640 supplemented with 15% heat-inactivated fetal bovine serum (FBS), 100 U/ml penicillin, 100 mg/ml streptomycin, 2 mM L-glutamine. After 24 hr fresh RPMI1640 medium was added without FBS (All from Life Technologies, Grand Island, NY, USA). Conditioned media were collected 24 hr later.

### Generation of Mo-DCs

3–5 ml apheresis of sample was collected in sterile bottles containing preservative-free heparin (40 IU of heparin/ml of sample). Mono-nuclear cells (MNCs) from the sample were re-suspended in Iscove’s modified Dulbecco’s medium (IMDM, Sigma Aldrich, St. Louis, MO, USA). MNCs were either cryopreserved (freezing medium contained 10% DMSO v/v and 20% FBS in IMDM) or used fresh. Monocytes were enriched by 1 hour plastic adherence and 10^7^ MNCs were seeded in 6 well plate (Corning-falcon, New York) per ml IMDM supplemented with 1% AB + plasma per well. After incubation adherent population was washed with plain IMDM to remove any contamination with undesired cells such as RBCs. After viable count adherent cells were set for differentiation in IMDM supplemented with 5% AB + plasma, GM-CSF (50 ng/ml) and IL-4 (30 ng/ml) for three days followed by GM-CSF (50 ng/ml) and TNFα (50 ng/ml) for four days. DCs were subjected to maturation by addition of TNFα, LPS and CD40L (100 ng/ml each) for 48 hrs^40^.

To examine the effect of IL6 on HD-DCs, exogenous IL6 (50ng/ml) was added in the healthy sample monocyte DCs culture at the day 0 and day 3. Rest of the differentiation and maturation was performed as mentioned earlier.

In yet another experiment Mo-DCs were differentiated in presence of 50% v/v U266B1 conditioned medium.

All recombinant human cytokines were procured from Peprotech Asia (Revohot, Israel).

### Morphological analysis of DCs

Mature HD-DCs and MM-DCs were observed for their morphology and adhered cells were stained with Wright’s and Giemsa Stain. Images were acquired on Olympus IX70 (Tokyo, Japan).

### Antigen uptake assay with Dextran-FITC

Receptor mediated endocytosis is a property of immature DCs. On day 5 of *in vitro* DC culture, cell were harvested and used for antigen uptake assay. Cells were suspended in PBS containing 1% FBS and FITC-dextran (20 mg/mL) (molecular wt. 40 kDa, Sigma Aldrich) and incubated either at 4 °C (internalization control) or at 37 °C, for 30 and 60 minutes. Cells were washed and acquired on FACS canto II (BD).

### Antigen uptake assay by using confocal microscopy

U266B1 cell line was labelled with PKH26 fluorescent dye (Sigma Aldrich) according to manufactures instructions. Cell lysis of PKH26 labelled U266B1 cells was carried out as described earlier by Rainone V. *et al*.^41^ with some modifications^41^. Briefly, harvested cells were washed with 1X PBS and heat-shocked at 42° for 1 hr and then incubated at 37° for 2 hrs. Mechanical disruption of cells was carried out by 5 cycles of rapid freeze and thaw. Cell debris was removed by centrifugation at 12,000 rpm for 15 minutes. Lysate of labelled U266B1 cells was administered to immature HD-DCs and MM-DCs for 24 hrs (Pulsed, U266B1 to DC ratio 5:1). After incubation DCs were stained for Phalloidin-FITC (Invitrogen) to identify cells and counterstained with DAPI (5ng/ml, Sigma Aldrich). DCs without any addition of labelled cell lysate were used as control (unpulsed). Stained DCs were acquired on LSM 510 Zeiss confocal microscope (Gottingen, Germany) and images were analysed using LSM Image Browser software.

### *In vitro* migration assay

CCL19 is a chemo attractant used to study DC migration through 8 µm plain cell culture inserts (BD Falcon™ Cell culture inserts). 10^4^ DCs in plain IMDM were loaded per culture insert and kept over wells containing 500 µL of IMDM with or without CCL19 at 500ng/mL (Peprotech). The cultures were incubated for 3 hours at 37 °C in 5% CO_2_. Cells migrated in lower chamber through the insert were harvested and viable cells were counted after trypan blue staining.

### *In vivo* DC migration

This assay was performed on the NOD/LtSZ-scid/scid strain of mice. They were obtained from the Jackson Laboratories and were bred in-house in the animal facility of NCCS. The study was conducted in accordance with the institution’s guidelines for animal husbandry and has been approved by IAEC-NCCS/CPCSEA (Institutional animal ethical committee-NCCS/Committee for the Purpose of Control and Supervision of Experiments on Animals). Approval number: IACUC/EAF/2013/B-199. HD-DCs and MM-DCs were labelled with Carboxyfluoresceinsuccinimidyl ester (CSFE, Molecular probes) according to the manufacturer’s protocol. Single cell suspension of labelled HD-DCs or MM-DCs was prepared in HBSS. A total of twelve 4–6 week old female NOD-SCID mice were used for the experiment. They were randomly segregated into two groups. One group of mice (n = 6) received HD-DCs from one sample and second group of mice (n = 6) received MM-DCs from other sample. 10^6^ DCs were injected subcutaneously (s.c.) per mouse in the groin region. Regional lymph nodes were harvested after 48 hr of DC injection, and the DCs migrated to the lymph nodes were quantitated by estimating human CD11c positive cells in gated CFSE positive cells using flow cytometry.

### Mo-DCs and T cell co-culture

To test the allo-stimulation of HD-DCs and MM-DCs, we performed co-culture of DCs with allogeneic T cells isolated from peripheral blood of healthy volunteers. From 10 ml of peripheral blood, CD3+ T cells were sort purified. DCs and T cell co-culture was performed in the ratio 1:10 for 5 days. After incubation, cells were stained with CD8-PE, CD4-PE and CD25-APC (BD Pharmingen).

In another set of experiments, CD3+ T cells were labelled with 5 µM CFSE (Molecular probes) for 10 mins. DCs and T cells were co cultured in a ratio of 1:10 for 5 days in 96 well plate. After incubation, T cells were counted using trypan blue staining. While counting, T cells were distinguished from DCs on the basis of size and morphology. CFSE dilution on T cells was determined by flow cytometry^42^.

### ELISA

Supernatants from HD-DCs, MM-DCs cultures and DC-T cell co-cultures were harvested by centrifugation and were frozen at −20 °C. The supernatants of HD-DCs and MM-DCs were subsequently assayed for cytokine content hIL-10 (OptEIA kit, BD Bioscience PharMingen) and hIL-12p70 (eBioscience) by sandwich ELISA as per the manufacturer’s instructions. The supernatants of DC-T cell co-cultures were assayed for hIFNγ using OptEIA kit.

#### Flow cytometry, gene expression by PCR and immunofluorescence analysis

See Supplementary methods.

### Statistical analysis

Data were analysed by Sigma Plot 11 software using one-way repeated measure analysis of variance (Jandel Scientific Software, California, USA). p value ≤ 0.05 (*) was considered significant and the graphs represent mean ± standard error of mean (SEM). ‘N’ represents number of samples on which the experiment was repeated and ‘n’ represents the number of replicates in one experiment.

### Data availability statement

The datasets generated during and/or analyzed during the current study are available from the corresponding author on reasonable request.

## Electronic supplementary material

Supplementary Information
