# Supplementary material for: Compromised functionality of monocyte-derived dendritic cells in multiple myeloma patients may limit their use in cancer immunotherapy
Source: Sci Rep. 2018 Apr 9;8:5705. doi: 10.1038/s41598-018-23943-w (PMC5890285; doi:10.1038/s41598-018-23943-w)
Supplement: Supplementary file 1 — Supplementary Information [file 41598_2018_23943_MOESM1_ESM.pdf]

# Compromised functionality of monocyte-derived dendritic cells in multiple myeloma patients may limit their use in cancer immunotherapy

Prajakta Shinde, Sophia Fernandes, Sameer Melinkeri, Vaijayanti Kale, Lalita Limaye*

## Supplementary information

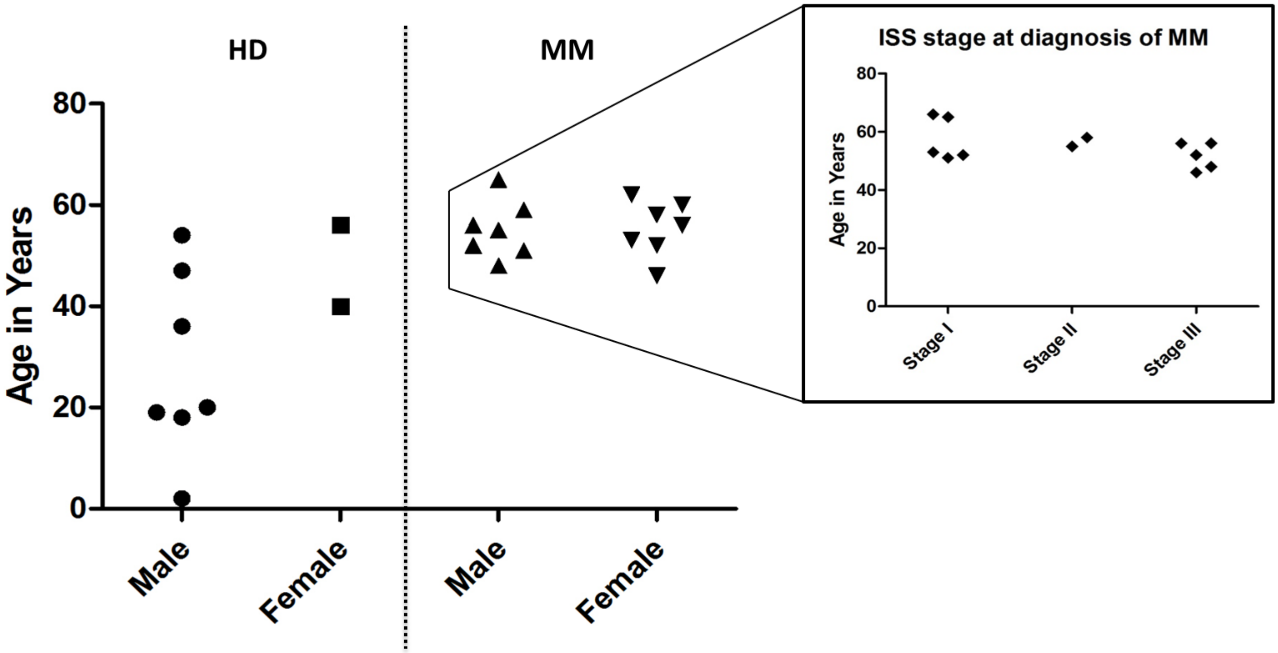


**Supplementary Figure S1:** Characteristics of Healthy donor (HD) and Multiple myeloma (MM) patients.

| Sample | Number of cell migrated $x10^3$ |
| --- | --- |
| HD 1 | 8.4 ± 0.30 |
| HD 2 | 7.4 ± 0.37 |
| HD 3 | 7.2 ± 0.51 |
| MM 1 | 6 ± 0.11 |
| MM 2 | 5.9 ± 0.15 |
| MM 3 | 4.4 ± 0.57 |

**Supplementary Table S2: Migration of HD-DCs and MM-DCs toward CCL19:** Migration of DCs from 3 HD and 3 MM samples.

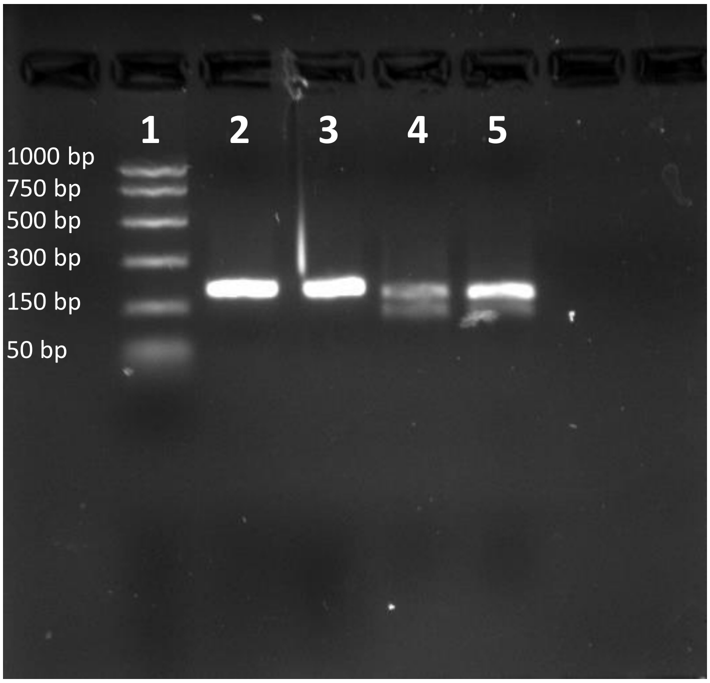


Lane 1: DNA ladder
Lane 2: GAPDH, HD
Lane 3: GAPDH, MM
Lane 4: IL6, HD
Lane 5: IL6, MM

**Supplementary Figure S3:** Representative full-length, PCR gel image of mRNA expression of GAPDH and IL-6 on HD and MM samples on day3 of DC culture

# Supplementary methods

## Flow cytometry analysis

Mature Mo-DCs were washed with 1% PBS (Phosphate Buffer Saline) and blocked in 1% BSA. Anti-human monoclonal antibodies namely FITC-conjugated HLA-DR, HLA-ABC and CD58; APC-conjugated CD1a, CD11c and CD40; PE-conjugated CD54, CD80, CD83 and CD86 were used for phenotyping of DCs (BD Pharmingen, San Diego, California, USA). Appropriate isotype controls were kept and cells were incubated on ice for 45 minutes. For assessment of pP38, day0 and day3 cells were harvested and treated with BD Fixation and Permeabilization reagent for 10 min on ice. Cells were washed in 1× wash buffer (BD) and purified pP38 (pT180/pY182, BD) antibody was added to the cells and incubated for 60 minutes on ice. This was followed by incubation with specific secondary antibody conjugated with Alexa Fluor 594 (Invitrogen). Stained cells were washed and 10,000 events were acquired on BD FACS canto II (BD) and data analysed by FACS DIVA, version 5.0 (BD) and Flow Jo software (LCC, Ashland OR, USA)

## Immunofluorescence analysis

Mature HD-DCs and MM-DCs smear was prepared using Shandon cytospin at 500 rpm for 3 min then fixed with 1% paraformaldehyde (PFA). Blocking was done with 1% BSA followed by staining with purified anti-CCR7 (BD) antibody. After incubation for 1 hr at room temperature, specific secondary antibody conjugated with Alexa Fluor 488 (Invitrogen) was used. Images were taken on LSM 510 Zeiss confocal microscope. Fluorescence intensity was calculated using ImageJ software.

**Gene expression analysis by real-time PCR**

Total RNA was extracted from cells using the Trizol reagent according to the manufacturer’s instructions (Invitrogen) and quantitated using Nanodrop spectrophotometer (ND1000). 1-μg RNA was reverse transcribed into cDNA using MMLV reverse transcriptase (Invitrogen). Real time PCR was carried out using the SYBR-Green PCR master mix on 7500 ABI-prism sequence detection system (Applied Biosystems, Foster City, CA). Negative controls comprised of samples without reverse transcription step. Glyceraldehyde-3-phosphate dehydrogenase (GAPDH) was used as a reference gene. Following is the list of primers used-

| No. | Gene name | Sequence (5′ → 3′) |
|---|---|---|
| 1 | Human GAPDH forward | CGG ATT TGG TCG TAT TG |
| 2 | Human GAPDH reverse | GGA AGA TGG TGA TGG GA |
| 3 | Human CCR7 forward | GGT ATG CCT GTG TCA AGA TG |
| 4 | Human CCR7 reverse | GGT TGA GCA GGT AGG TAT CG |
| 5 | Human IL6 forward | CAA TGA GGA GAC TTG CCT GG |
| 6 | Human IL6 reverse | TGG GTC AGG GGT GGT TAT TG |
